# Remaining Useful Life Prediction Based on Deep Learning: A Survey

**DOI:** 10.3390/s24113454

**Published:** 2024-05-27

**Authors:** Fuhui Wu, Qingbo Wu, Yusong Tan, Xinghua Xu

**Affiliations:** 1 School of Information Engineering, Wuhan College, Wuhan 430212, China; 2 College of Computer, National University of Defense Technology, Changsha 410073, China; 3 National Key Laboratory of Science and Technology on Vessel Integrated Power System, Naval University of Engineering, Wuhan 430033, China

**Keywords:** remaining useful life prediction, deep learning, survey

## Abstract

Remaining useful life (RUL) is a metric of health state for essential equipment. It plays a significant role in health management. However, RUL is often random and unknown. One type of physics-based method builds a mathematical model for RUL using prior principles, but this is a tough task in real-world applications. Another type of method estimates RUL from available information through condition and health monitoring; this is known as the data-driven method. Traditional data-driven methods require significant human effort in designing health features to represent performance degradation, yet the prediction accuracy is limited. With breakthroughs in various application scenarios in recent years, deep learning techniques provide new insights into this problem. Over the past few years, deep-learning-based RUL prediction has attracted increasing attention from the academic community. Therefore, it is necessary to conduct a survey on deep-learning-based RUL prediction. To ensure a comprehensive survey, the literature is reviewed from three dimensions. Firstly, a unified framework is proposed for deep-learning-based RUL prediction and the models and approaches in the literature are reviewed under this framework. Secondly, detailed estimation processes are compared from the perspective of different deep learning models. Thirdly, the literature is examined from the perspective of specific problems, such as scenarios where the collected data consist of limited labeled data. Finally, the main challenges and future directions are summarized.

## 1. Introduction

Remaining useful life (RUL) is the useful life left in an asset at a particular time of operation. It is a crucial technology in health management. Accurate RUL prediction provides instructions for system design, production, and maintenance. For device maintenance, its costs constitute a large portion of the operating and overhead expenses in industries. Hence, both academia and industry are committed to promoting RUL prediction technology for better maintenance strategies.

Generally, there are three strategies for performing maintenance [1,2]. The first strategy is to perform maintenance after the occurrence of failure. Under this strategy, assets experience the least number of maintenance events during their entire life cycle. However, it often shortens the life span of assets due to irreversible damage caused by failure. The second strategy is to perform maintenance periodically, known as time-based maintenance (TBM). This preventive maintenance strategy assumes that the estimated mean time between failures is statistically or experientially known. The third strategy is called condition-based maintenance (CBM). It utilizes component-specific sensors to monitor the health information of a component. A component’s degradation state is evaluated based on deviations from normal running conditions. However, the deviations may not necessarily equate to failure in case of operating condition changes or acceptable degradation levels. By establishing a relationship between the reliability characteristics of a component’s population and sensor signals, a more accurate assessment of the degradation process can be made. Unlike the periodical maintenance of TBM, the CBM strategy aims to perform maintenance at the optimal time point based on collected condition information. Considering the advantages of CBM, it is currently a hot topic in the literature.

When talking about CBM, diagnostics and prognostics are two important aspects that should be distinguished. Diagnostics deals with fault detection, isolation, and identification when abnormalities occur, while prognostics deals with fault and degradation prediction before they occur. As diagnostics has already been widely studied and applied in industry. The prognostics aspect which has not made significant progress is focused on in this survey. It is an active area of research and development in the aerospace, automotive, nuclear, process controls, and national defense fields [3]. Among all prognostic technologies, remaining useful life (RUL) estimation is a key technology, which is also useful in other fields, such as resource recycling [4].

In order to precisely estimate remaining useful life, methods are usually divided into two categories: model-based methods and data-driven methods. Because it is usually a tough task to accurately build a mathematical model for a physical system using prior principles in real-world applications, the data-driven model is currently the main method being researched. It discovers the law of performance degradation from history trajectories and forms the RUL model.

Traditional data-driven RUL prediction methods include least-squares predictor model and autoregressive moving-average (ARMA) model. They usually consist of two steps. Firstly, features are extracted from the input data. Then, these features are mapped to an RUL value. The bottleneck of this kind of method lies in designing suitable health features, which is highly manpower-intensive but has limited prediction accuracy. To address this issue, shallow neural networks [5,6,7] are adopted, but their performance still does not meet the demands of real-world applications. Traditional data-driven RUL prediction methods can be classified into two broad types: models based on the directly observed state processes and those based on unobservable state processes [8]. The former includes the regression, Brownian motion, Gamma processes, and Markovian-based models. The latter includes stochastic-filtering-based models, covariate-based hazard models, and methods based on hidden Markov models (HMM) and hidden semi-Markov models (HSMM). Analysis of traditional data-driven methods reveals four common shortcomings and challenges: (i) the reliance on physics-based models, (ii) data fusion where multidimensional input data must be handled, (iii) modeling the influence of external environmental variables, and (iv) the development of a model that can handle multiple failure modes.

In recent years, deep learning technology has made great progress in image identification, machine translation, autopilot, and other areas. This provides new ideas for RUL prediction. Deep learning technology enables the establishment of end-to-end models from historical data to RUL value, avoiding the complex feature extraction process. Moreover, the working environment of assets whose RUL is to be predicted is usually complex and noisy. The deep learning method is able to handle these problems automatically without field knowledge.

Another significant factor behind this trend is the big data paradigm. Traditional data-driven RUL prediction methods cannot cope with the big data scenario, while deep learning methods are naturally developed for the big data paradigm. When applying deep learning to big data processing, it is able to learn multi-scale, multi-level, and hierarchical representation knowledge. This makes it inherently feasible for data-driven RUL prediction.

**Figure 1:**
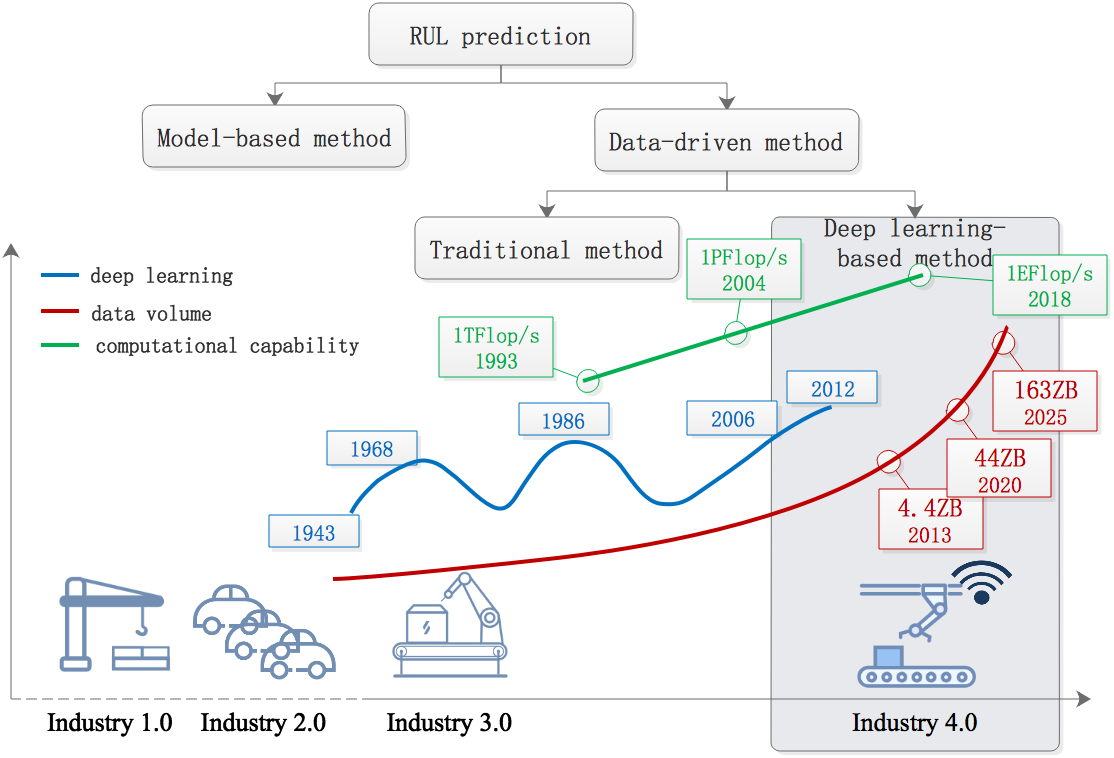
The development background of RUL prediction based on deep learning.

Taking a look at the technical state, it is observed that all key aspects are prepared for adopting deep learning methods for RUL prediction. Currently, we are experiencing the fourth industrial revolution, where accurate RUL prediction is an urgent need. A typical characteristic of “Industry 4.0” is intelligentization, which relies on big data, computational capability, and algorithm models. These three aspects are intertwined and develop together, as shown in Figure 1.

In the context of the industrial internet of things or Industry 4.0, massive sensor data collected forms the big data for RUL prediction. Since the 1980s, the ability to store information per capita worldwide has doubled every 40 months [9]. According to a report by IDC, the scale and growth rate of data in the world is shown as the data volume curve in Figure 1. This phenomenon has spawned the fourth paradigm of scientific research, known as data-intensive science in the era of big data [10].

Deep learning has gained a new opportunity for rapid development in this big data context. After several peaks and valleys, the development of deep learning technology has reached a stage of widespread use. It originated from simple models such as the MP model and Hebb’s rule, and the first truly meaningful two-layer neural network perceptron was published in 1958. Benefiting from new discoveries in human visual system, the first deep learning system of a feedforward multi-layer neural network was trained using the Group Method of Data Handling (GMDH) method [11] in 1968. Then, the neural network entered the first “ice age”. In 1982, John Hopefield proposed a recurrent neural network that simulates human memory, restoring the vitality of the neural network to some extent. Four years later, the back-propagation method solved the problem of computational power that caused the first “ice age”, marking the second rise of deep learning [12]. In 2006, Professor Hinton firstly proposed a deep belief network, defining the method of combined learning by multi-layer neural networks as deep learning [13]. This marked the third rise of deep learning. Since then, deep learning has made major breakthroughs in the fields of speech recognition and image recognition, etc.

The history of deep learning reveals that computational complexity is an important factor limiting the development of deep learning. From the high performance computing Top500 (www.top500.org) website, it is observed that we have entered the exascale computing era (E-level) after the terascale (T-level) and petascale (P-level) calculations, as shown in Figure 1. This promotes the development of deep learning. On the other hand, it also provides the basis for big data analysis.

Hence, the problem of RUL prediction is expected to achieve breakthroughs with deep learning methods. Some acronyms used are summarized in Table 1.

## 2. Related Works

In the literature, there are already several surveys on related works. In 2007, Schwabacher et al. [14] carried out a survey on artificial intelligence methods for prognostics. It was tied to a real-world project on NASA’s on-board Integrated Systems Health Management (ISHM) system. As described above, RUL prediction methods are classified into two broad types: model-based and data-driven methods. In 2010, Si et al. [8] reviewed statistical data-driven approaches. They suggested that past recorded failure data may be scarce because critical assets are not allowed to run to failure. However, a broad sense of any data, which they named CM data, is a more important source of information. They further classified the observed CM data into direct CM and indirect CM data. Keeping this idea in mind, they surveyed direct and indirect CM data-driven approaches, respectively. Liu et al. [15] reviewed artificial intelligence approaches for the fault diagnosis of rotating machinery. Nash et al. [16] surveyed deep learning technology in the study of materials degradation. They classified the detection of degradation into direct and indirect detection. Zhao et al. [17] provided a review of fault diagnosis and prognostic methods based on deep learning technology. They focused on deep neural network (DNN), deep belief network (DBN) and convolutional neural network (CNN) models, and summarized some potential future research issues. Khan et al. [18] presented a systematic review of deep-learning-based health management systems. They focused on three deep learning architectures: auto-encoder, convolutional neural network, and recurrent neural network. Zhao et al. [19] reviewed the application of deep learning technology to machine health monitoring from the perspective of auto-encoder, CNN, and RNN models. Moreover, experiments were conducted to study the performance of these approaches. Surveys that focus on deep-learning-based RUL prediction were conducted by Remadna et al. [20] and Wang et al. [21]. However, a limited number of works in the literature were reviewed in [20], and only lithium-ion batteries are considered in [21].

An overall comparison between related surveys is summarized in Table 2. They are compared in two dimensions of goal and method. The goal dimension is divided into PHM and RUL prediction. The goal of PHM is wider. It contains not only prognostics but also diagnostics. In a PHM system, selected parameters are monitored. Once anomaly is detected, the diagnostic process will be carried out to locate it. Prognosis, however, is a process aimed at predicting and estimating the RUL of a system. In the method dimension, related surveys are classified into three types: TDD, SNN, and DNN. The abbreviation of TDD denotes the traditional data-driven method, while SNN and DNN denote the shallow neural network method and the deep neural network method, respectively. It is important to note that both the SNN method and the DNN method belong to the data-driven category. In this survey, only the DNN method for the RUL prediction problem of prognostics is focused on. Throughout this survey, it is observed that RUL prediction and deep learning technology are both hot research topics. RUL prediction is promoted by industry needs, and deep learning is considered a revolutionary technology. Most works that focus on using deep learning methods in RUL prediction were carried out in the past several years. At present, it is necessary to conduct a survey on the challenge, approach, and future of deep-learning-based RUL prediction.

The following are four principles that guide this survey:

1. The literature is reviewed under unified problem formulation and framework for deep-learning-based RUL prediction.
2. Different from deep neural network surveys, related works are surveyed mainly from the perspective of the RUL prediction problem.
3. Details of deep technology are not introduced in the paper. It is assumed that readers already have this knowledge or can acquire it from other sources.
4. In order to study the general methodology of deep-learning-based RUL prediction, specific application fields or test datasets will not be detailed.

## 3. Unified Framework and Models

### 3.1. Problem Formulation

Currently, there is no clear definition of remaining useful life (RUL). It is even difficult to define the failure time of a system. Generally, the remaining useful life is defined as the time length from current time point to the failure time point intuitively.

 **Definition 1.**  *T: Let T be the time of the system failure.*

 **Definition 2.**  *t: Let the system has survived until time t.*

From the perspective of statistics [22], the remaining useful life (RUL) can be defined as:

(1) rul=T−t

Then, the deep-learning-based RUL prediction method constructs a deep neural network that calculates the RUL value from raw input data in an end-to-end manner.

### 3.2. Unified Framework

**Figure 2:**
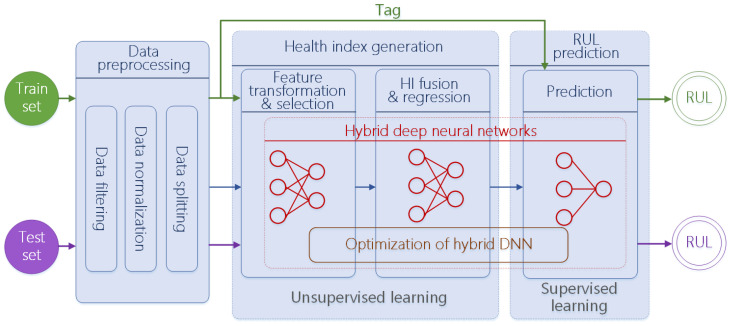
Unified deep-learning-based RUL prediction framework.

A unified framework of deep-learning-based RUL prediction is shown in Figure 2. It covers most related works in the literature. A general deep-learning-based RUL prediction method is made up of three stages. At the data preprocessing stage, collected raw data are preprocessed by filtering, normalization, and splitting. The preprocessed data are suitable as the input of deep neural network. At the health indicator generation stage, it aims to generate features with strong representation ability of health. At the last stage, it performs prediction and outputs the final RUL value. In a specific scenario, it is not always necessary to combine all stages. In some works, they generate RUL values just at the health indicator fusion and smoothing step. Furthermore, some methods directly feed collected data into RUL prediction deep neural networks.

#### 3.2.1. Data Preprocessing

Currently, systems are usually composed of various interacting components, in which the collective actions are difficult to deduce from those of the individual elements. This leads to the result that predictability is usually nonlinear. For condition monitoring, there are usually multiple sensors that receive information about system health due to incomplete understanding of the multidimensional failure mechanisms. Along with data noise, these factors render current methods ineffective. Moreover, real-world systems usually operate under a variety of operating conditions. All these situations make the data preprocessing step important for accurate RUL prediction.

In deep-learning-based RUL prediction, data preprocessing involves three aspects: data filtering, data normalization, and data splitting.

(1) Data filtering

In real applications, operations and working conditions are usually complicated. It is hard to obtain an optimized sensor deployment strategy. Deep learning models may not fully grasp the underlying physical processes solely by using raw input data. The successful prognosis of remaining useful life requires proper filtering of raw data. Generally speaking, selected data should possess abilities of diagnostic, the sensitivity, and consistency. The diagnostic ability is the basic metric that determines the prediction accuracy of selected data. The sensitivity metric is highly correlated with prediction performance. Furthermore, the consistency metric is related to the confidence of the predicted result.

The collected data usually vary in sensitivity to both spatial and temporal spaces. As the bearing vibration signal, for example, time domain features are insensitive to small changes but sensitive to noise. They have an advantage in representing the middle stage of the bearing degradation process, while the frequency domain features are sensitive to the earlier and later stages [23]. The filtering of input signals requires a comprehensive consideration of degradation pattern representational ability, information redundancy, and complexity of signal features.

In order to enhance the diagnostic ability of collected data, Wu et al. [24] designed a strategy to prepare additional input data before training, which is referred to as dynamic differential data. These data are calculated as the forward difference between the current sensor monitoring value and the previous value under the same operation mode. The dynamic differential data inherently contain a great amount of information about the performance degradation of an asset. Based on this strategy, they proposed a vanilla LSTM neural network for RUL prediction.

(2) Data normalization

In many cases, raw inputs differ in scale. Directly feeding these data into a deep learning network will lead to unequally weighted input features and will slow down the learning and convergence of model training. Therefore, raw inputs are usually converted into a common scale. The most common technique is z-score normalization [25]. Bektas et al. [26] proposed a component-wise multi-regime normalization method for data preprocessing to provide a common scale across all the complex features.

(3) Data splitting

When splitting data for a deep learning model, there are three levels to consider.

Firstly, datasets are split into training datasets and test datasets. Special data splitting strategies can optimize the hyperparameters of deep neural networks and calculate the uncertainty of the deep learning model as well. Labeled data and unlabeled data are prepared for supervised learning and unsupervised learning separately. They can also be combined as semi-supervised learning.

Secondly, the full life cycle of an asset is usually split into the health stage and the deterioration stage. Furthermore, the selection of the deterioration start point is sensitive to the performance of prediction models. The performance of most assets usually degrades after a certain period of usage after being produced, but does not following a linear trend. The beginning part of a data sequence is designated as healthy part. However, it is hard to choose the degrading point, which is a common issue in the literature. Most research in the literature assumes that the degradation is not noticeable, and the RUL will be piecewise continuous, such that the RUL is constant at the beginning and then decreases linearly. However, this assumption often makes these models impractical for real-world tasks. Heimes et al. [27] proposed a multi-layer perceptron neural network (MLP) classifier, trained by the extend Kalman filter (EKF) [28], to distinguish between degraded and healthy status data. Li et al. [29] proposed an approach to determine the first prediction time (FPT) based on kurtosis [30]. Based on this feature, the prediction process is separated into two stages. The first stage is to predict the degradation state of the system, and the sensitive features from the first stage are then fed into the second stage to predict the remaining useful life [31,32].

Thirdly, in accordance with different data splitting methods, there are three mapping modes from input data to output value. They are the point-to-point (P2P) mode, the sequence-to-point (S2P) mode, and the sequence-to-sequence (S2S) mode. The P2P calculates an RUL value for every input record. The S2P generates an RUL value from a sequence input record, while the S2S will obtain more than one output value. For the RUL prediction scenario, P2P and S2P are usually adopted. Lim et al. [33] studied the effect of splitting time window size on prediction accuracy. Elsheikh et al. [34] chunked training sequences into consecutive overlapping windows of observation sequences. Each of these input sequences is fed into a bidirectional handshaking LSTM (BHSLSTM), and the output is the corresponding RUL. These windows allow the network to experience observation sequences from systems starting at different initial health states, which avoids the aforementioned problems.

Then, a common question left is how to prepare input data for deep learning neural networks. There are three general categories of point-wise, segment-wise, and temporal-wise methods. A variation of the segment-wise method is the time window sliding approach [25]. The goal of an efficient data splitting mechanism is to enhance the generalization power and training performance of a model. Wang et al. [35] proposed a hybrid long–short sequences method for engine RUL prediction. LSTM architecture is used for RUL predicting of long temporal sequences. Furthermore, gradient-boosting regression (GBR) is used to predict RUL of time window sequences. The RUL outputs of both LSTM and GBR are fed into a back-propagation neural network to obtain the final prediction.

#### 3.2.2. Health Indicator Generation

The health of a system is complicated. There usually does not exists a deterministic indicator to express the health state. In order to calculate the RUL value, a metric of health indexing (HI) is generated first to represent the health state. Three key properties of HI are monotonicity, trendability, and prognosability. Monotonicity requires that the equipment does not undergo self-healing, which would result in non-monotonic trends. Trendability indicates the degree to which the evolution of the health indicator has the same shape and can be described by the same functional form. Prognosability measures the variance of the HI values at failure time. It is expected that failures occur at the same value of the HI.

Because of the unmeasurable feature of health indexing, datasets for deep neural network do not have health indicator training labels. This stage is usually implemented using unsupervised learning methods.

The health indicator generation stage can be further divided into two steps of feature transformation and HI fusion.

(1) Feature transformation and selection

Different from feature extraction of traditional data-driven method, where features are designed based on a physical model, feature transformation is implemented using deep learning methods. It maps input data to feature spaces with strong correlations to the health state. In this process, noise reduction is also performed.

(2) HI fusion and regression

Extracted features usually contribute different weights to the final health indicator. Compared with traditional health indicator generation methods, deep-learning-based methods can adaptively complete health indicator fusion. Moreover, deep learning methods are able to discover the knowledge of health indicators at different scales. After HI fusion and regression, the generated health indicator is prepared to predict the RUL value.

#### 3.2.3. RUL Prediction

At this stage, it performs the final prediction using deep learning methods. It is critical to choose a suitable deep neural network model. The deep neural network is trained and optimized for high prediction performance and generalization ability.

(1) Deep neural network model

In the application of RUL prediction, the two most widely used models are the recurrent neural network (RNN) and the convolutional neural network (CNN). The first one is able to discover temporal features, and the second one is able to discover different scale features. In order to discover more features, different deep neural networks can also be combined to predict the RUL value.

(2) Deep neural network training

To train a deep neural network, some common problems include over-fitting, under-fitting, gradient explosion, and gradient disappearance. Accordingly, the solution is to design proper activation functions, loss functions, and regularization strategies. Despite this, there are situations that general training methods cannot handle. Special methods, such as cross-validation and transfer learning, may be adopted.

(3) Deep neural network optimization

The main optimization goal here is met using the hyperparameters of the deep neural network. It is a complicated problem, whose objectives include enhancing prediction performance and improving convergence for predicting RUL using deep learning methods. A general method is to treat it as a multi-objective optimization problem and design a proper algorithm to search for globally optimized parameters.

## 4. Application of Deep Learning in RUL Prediction

Based on the proposed unified framework in Section 3, the mechanism of how deep learning technology is applied for RUL prediction is reviewed. The applications are categorized into four types: deep-learning-based health indexing, deep neural network model towards applications, deep learning method applications, and ad hoc deep learning applications for RUL prediction. As the deep learning method aims to implement RUL prediction in an end-to-end manner, without relying on human labor for feature design, a new approach is needed to establish health indices. Hence, the first problem is working out how to utilize deep learning technology to implement health indexing. Then, the application of deep learning to RUL prediction is reviewed from the perspective of different deep neural network models. In order to complete the final step of application in a production environment, deep learning methods such as transfer learning, hybrid deep learning, and ensemble learning are adopted. Additionally, ad hoc deep learning approaches are devised to address common challenges encountered in practice.

### 4.1. Deep-Learning-Based Health Indexing

Conceptually, health indexing involves obtaining metrics that can quantitatively evaluate the health status of assets. Efficient health indexing metrics should possess properties of monotonicity, trendability, and prognosability [36,37]. Generally, health indicators (HIs) are categorized into physical HIs (PHIs) and fused HIs (FHIs). PHIs refer to the extraction of physical significantly related information from the acquired data using statistics or signal processing algorithms. FHIs, also known as synthesized HIs or virtual HIs, are typically constructed using multiple PHIs or multiple sensor information via data fusion algorithms. In real-world applications, PHIs are usually difficult to obtain due to limited knowledge about physical properties. Recently, synthesized HIs have received significant attention [38].

FHIs are usually constructed by manually fusing multidimensional statistical features. They have already been reported to have achieved good results in the literature. However, synthesized HIs still have three drawbacks. Firstly, statistical features have different ranges, and these features do not contribute equally to the construction of HIs. Secondly, it is difficult to determine a definite failure threshold because HI values have a large variation range among different assets at the time of failure. Thirdly, statistical features vary in their sensitivity to faults. Furthermore, there are often multiple components that interact with each other in a complex system, resulting in intricate dependencies between sensor data.

Feature transformation and fusion are two essential steps in obtaining a health indexing model that satisfies three properties of a health indicator. The essence of feature transformation is to reduce the dimension of raw features. Then, a denoising process is adopted to smooth local random fluctuations and enhance the monotonicity of the health indicator. Table 3 summarizes health indexing technologies used in the literature.

#### 4.1.1. Feature Transformation and Selection

There are two functionalities of feature transformation. The first is to transform raw data into features that are most related to health status [46] and are feasible for subsequent processing steps. The other is to transform features in one space to features in another space. The objective of space transformation is dimension reduction. To implement the first functionality, some common methods include Fourier transform (FT), Wigner–Ville distribution (WVD), wavelet transform (WT), and continuous wavelet transform (CWT) [45] in time, frequency, and time–frequency domains. Because the handcrafted feature extraction approach is highly dependent on expert prior knowledge and labor resources, deep learning methods are leveraged to address this problem. Generally, it is impossible to obtain correct values of features, so deep learning in this step usually belongs to the unsupervised learning method. The auto-encoder (AE) [49], including its variations of enhanced auto-encoder (EAE) [42], stacked denoising auto-encoder (SDA) [43,44], and stacked sparse auto-encoder (SAE) [45], are able to automatically extract highly abstracted features. For feature extraction, the convolutional neural networks (CNNs) are able to take advantage of the spatial structure of input data, especially in vibration signal processing scenarios, through local connections and pooling [40]. In order to take advantage of temporal characteristics of input data when performing feature transformation, the RNN can be adopted. To enable RNN model with denoising capability, it is extended as RNN encoder–decoder (RNN-ED). Furthermore, the masking vector and delta vector techniques can be adopted to handle value missing problems [47,48].

For the second functionality of feature space transformation, a classical technology is principal component analysis (PCA) [41]. PCA is a linear technique that maps original features into a new feature space with fewer dimensions and extracts principal components. Based on PCA, the kernel principal component analysis (KPCA) technology adds a step before PCA by employing nonlinear kernel functions to map the original features into a new hyper dimensional feature space [51]. Self-organizing map (SOM) is a neural-network-based clustering method that is able to map features in higher-dimensional space into features in lower-dimensional space without changing the top structure. This capability is feasible for feature transformation [42].

As transformed features can be various and correlated, only part of them will be selected. The selection criteria should satisfy the requirement of an efficient health indicator [46]. As mentioned previously, there are three properties of a health indicator. The selection of feasible features can be taken as a multi-objective optimization problem [39]. It is important to note that the selection is strongly correlated with the second stage of fusion, where the aim is to select features that are ultimately beneficial for generating an efficient health indicator.

#### 4.1.2. Health Indicator Fusion and Regression

After transformation and selection, features are fused into a single health indicator (HI). The premise is that features that individually do not have the characteristics of health indicators may provide useful information when considered jointly. The straight forward idea is to calculate the time residual from the current time point to the failure point. Then, the health indicator fusion problem is transformed into a multidimensional distance calculation problem. Solutions, such as the AAKR approach, can be adopted [39].

Another idea for health indicator fusion is to implement it as a weighted average value, and the key technology is to design an efficient weighting mechanism. To address this problem, global search methods, such as the grey wolf optimizer (GWO) algorithm, can be adopted to calculate weighting coefficients [42]. For most deep-learning-based feature extraction and selection methods, the features are mapped to feed-forward single- or multiple-layer perceptron networks as a weighting approach to calculate the fusion health indicator [40].

Convolutional neural networks (CNNs) are a form of deep learning model that are able to implement feature transformation, selection, and fusion into a single process [52]. A typical CNN comprises three layers: the convolutional layer, the subsampling layer, and the fully connected layer. The structure of a CNN is a key factor that affects the accuracy of the generated health indicator [53]. Because the convolutional operation is able to handle multidimensional input, the time–frequency domain features extracted using continuous wavelet transform (CWT) are inherently feasible for CNNs. The recurrent neural network (RNN) is a deep neural network that is able to take advantage of mutual information from selected features. This makes it an option for health indicator fusion [46]. The long short-term memory (LSTM) is an RNN that is able to keep track of historical behavior features and combine those features with present measured features to fuse the final health indicator [45].

The problem that arises after fusion is health indicator regression, which is required to predict future health indicators. Theoretically, the health indicator changes monotonically. There may be outlier regions that affect the performance of the health indicator. The first step is to detect outliers, also known as trend burrs. Solutions include machine learning, inform theory, and statistics methods. For the statistics approach, the HI is treated as a normally distributed random variable. Then, a sample will be considered an outlier according to statistical rules, such as the 3σ rule [40]. In order to smooth the health indicator, moving average (MA) is a common approach for coping with time series trajectories [49]. It takes both the current observation and historical observations into account to estimate the current value. The exponentially weighted moving average (EWMA) [54] approach is a variation of MA that assigns exponential weight to historical observations, decayed by the distance from the current step.

Another more general problem is the nonlinear characteristic of health degradation. In this situation, deep neural networks such as hierarchical gated recurrent unit (GRU) are suitable for health indicator regression [41]. The Gaussian process regression (GPR) algorithm is a kernel-based machine learning technique that can conveniently specify high-dimensional and flexible nonlinear regression. It is used to estimate the future failure health indicator up to the current time [52]. It is important to note that an application does not have to follow a single regression model. On the one hand, the failure types and degradation patterns differ between different assets. In this scenario, clustering algorithms, such as K-means, can be adopted to separate assets and environments into different models [52]. On the other hand, the degradation patterns differ at different stages of the same asset. The regression models may differ at different stages [43,44].

### 4.2. Deep Neural Network Models for RUL Prediction

In this section, the application of deep learning to RUL prediction is reviewed from the perspective of different deep neural network models, including the auto-encoder, the restricted Boltzmann machine, the recurrent neural network, and the convolutional neural network models.

#### 4.2.1. Auto-Encoder Model

The most common usage of the auto-encoder (AE) model is feature learning in an unsupervised manner. In real applications, data often contain sparse features that are noisy and redundant in raw signals. Auto-encoders are naturally suitable for addressing these problems. To discover more information from raw input data, auto-encoders can be stacked to explore features in different scale layers. Then, the problem is how to train a stacked auto-encoder. Lin et al. [55] proposed the HELM [56] approach to solve the problem for the turbofan engines scenario, which is evaluated as being more efficient than back-propagation training algorithms.

Considering that collected data consist of time series, an accurate prediction requires a reasonable fusion of damage tendency and current states. Integrated deep denoising auto-encoders (IDDAs) can be combined to address this issue. The idea behind this design is that distant records are used to simulate the damage trend, while recent records are used to simulate the smoothing process of recent changes. Yan et al. [57] implemented an IDDA architecture with two deep denoising auto-encoders (DDAs) and a linear regression analysis. They split time series data into distant records and recent records at each time moment during the training phase. These two types of records are fed into the two DDAs, respectively. Based on this technology, they presented a concept of device electrocardiogram (DECG) to replace the traditional factory information system (FIS).

#### 4.2.2. Restricted Boltzmann Machine Model

Restricted Boltzmann machine is also a type of unsupervised machine learning algorithm. It is a generative stochastic artificial neural network that learns the probability distribution over input datasets. It is typically used for feature compression and denoising. To obtain RUL, it is usually followed by a supervised learning model [58]. Liao et al. [59] proposed an RUL prediction method following the two-stage paradigm. In the first stage, they developed an enhanced restricted Boltzmann machine to automatically generate features. To avoid over-fitting and stabilize the learning process of the enhanced RBM, they did not adopt traditional weight decay and sparsity regularization. Instead, they first designed a slope regularization term to represent the trendability. Then, the SOM algorithm [60] maps the high-dimensional output from the RBM to a one-dimensional health indicator. Based on the health indicator, RULs are predicted in a similarity-based manner (Table 4).

Through stacking multiple layers of RBMs, a deep belief network (DBN) is formed, which is able to learn the deep representation of the input data and is suitable for time series prediction problems [64]. The training of an unsupervised DBN is usually processed in a greedy, layer-wise manner. When applied to classification tasks, another discriminative learning procedure is implemented after the generative pretraining process of the DBN. This model is named the discriminative deep belief network (DDBN). The discriminative fine-tuning is carried out by adding a final layer of variables that represent the desired label samples. Ma et al. [61] proposed a discriminative deep belief network and ant colony optimization (DDBN-ACO) method for health status assessment for bearing and turbine engine machines. The ant colony optimization (ACO) technique is adopted to obtain optimal hyperparameters of the DBN. Wang et al. [62] investigated a deep-learning-based approach for material removal rate (MRR) prediction in polishing. Stacked RBMs are trained to represent input data in multiple high-dimensional spaces. Then, the output of stacked RBMs and the input are both fed to a feed-forward three-layer neural network for MRR regression. The network structure and learning rate are optimized through a particle swarm optimization (PSO) algorithm. The idea behind these two applications is the same, while the major difference lies in the structure. The first model takes the output of the last RBM as input for the fine-tuning network, while the second model takes outputs of multiple stacked RBMs as joint input for the feed-forward neural network.

One problem with the standard RBM with stochastic binary units is that it tends to model discrete data instead of continuous data. The continuous deep belief network (CDBN) is constructed with continuously valued stochastic units to solve this problem. Shao et al. [63] proposed a novel CDBN method for predicting rolling bearing performance degradation. It first uses locally linear embedding to quantify health degradation. Then, a continuous deep belief network (CDBN) is constructed based on a series of continuous restricted Boltzmann machines (CRBMs) [65] to learn the hidden nonlinear relationships. To optimize the learning rate and the number of units in the CDBN, they adopted the genetic algorithm (GA) as an intelligent optimization method.

#### 4.2.3. Recurrent Neural Network Model

The neural networks can be categorized into two major categories: feed-forward neural networks, which do not have feedback connections from a layer to a previous layer, and recurrent neural networks, where there are feedback connections. Generally speaking, recurrent neural networks are naturally suitable for time series prediction [66,67,68]. Hence, they are also the most widely researched models for RUL prediction applications. In turn, RNN methods in those applications are classified into four types according to the structures of RNN models (Table 5).

(1) Standard RNN methods

The most straightforward RNN structure is to link the output of an FNN to an extra input node, forming a feedback loop. Tse et al. [69] implemented this structure for time series prediction at the early stage. The experimental results claimed that FNN and RNN methods have better performance than conventional autoregressive models. Tian et al. [71] integrated two context layers from the Elman network and the Jordan network separately to construct an extended RNN model, which was confirmed to be more accurate than the Jordan network and the Elman network individually. Liu et al. [72] developed an adaptive RNN model for lithium-ion battery remaining useful life estimation. The predictor is constructed based on a feed-forward multi-layer neural network with adaptive and recurrent feedback links from output nodes and hidden nodes separately. The adaptive feedback links represent temporal information spatially, while the recurrent feedback links deal with time explicitly. Experimental results show that their method outperforms the classical RNN and the RNF.

For the training of RNN models, a primary issue is the selection between incremental and batch training mechanisms. In the long-term RUL prediction problem using the RNN model, Malhi et al. [70] proposed to use competitive learning for input data clustering before RNN training. After that, the recurrent neural network is batch-trained to calculate the initial weight. Then, it is incrementally trained for the final cluster. It is confirmed that the batch training is useful for trend prediction, which avoids the over-fitting associated with incremental training. To calculate parameters in order to obtain an accurate RNN model, truncated back-propagation through time (BPTT) [27] can be adopted to compute the gradients. Furthermore, the extended Kalman filter [28] training method can be utilized to update the weights of the network. Moreover, the differential evolution (DE) [102] process can be used to enhance the generalization power of RNNs and optimize over-fitting issues.

(2) ESN methods

The echo state network (ESN) is a paradigm of RNN that randomly establishes a large sparse reservoir to replace the hidden layers of RNNs. One of the main advantages of ESN lies in its training procedure, which is based on simple linear regression. It can be trained with little computational effort, while still providing the high generalization capabilities of RNN models. The ESN method was used to predict the degradation evolution of the stack for the proton exchange membrane fuel cell (PEMFC) [74,103]. When setting up the architecture of an ESN network, it is difficult to determine the optimal parameters. The multi-objective differential evolution method was adopted to search for globally optimized parameters for RUL prediction in ESN networks [104].

In order to improve the estimation precision for complex datasets with different features, an intuitive idea is to combine multiple ESNs that match to the varied datasets to address the problem. Subsequently, the Kalman filter technique is adopted to cope with multiple outputs of these ESNs. Peng et al. [73] proposed another combining ESN approach. Their approach classifies input data units into groups based on the condition data features, and trains a separate ESN model for each group, establishing an ESN model library.

(3) LSTM methods

The most serious problem of standard RNN is that the early time information cannot be retained through the network. To address this problem, long–short time memory (LSTM) [105] is the most widely used RNN, which is able to learn long-term patterns [91]. Moreover, the problems of gradient vanishing and exploding are also effectively addressed in LSTM compared to traditional RNN models. The application of LSTM for RUL prediction of C-MASS data and PHM 08 Challenge data can be found in [76]. Other application fields include PEMFC [77], hard disk drivers (HDDs) [90], lithium-ion battery [78,79,80,81], jet engines [84], aero engines [85], and rolling bearing [86].

As the input data pass through neural network, information generated in each layer can be regarded as representation of the input in a specific dimensional space. Adding additional layer could potentially unveil deeper relationships between the inputs and outputs. Hsu et al. [89] proposed a two-layer LSTM architecture for RUL prediction. Zhang et al. [91] and Zheng et al. [92] developed deep LSTM approaches by stacking multiple LSTM layers. Zhao et al. [93] also proposed a deep LSTM approach for tool wear prediction by stacking multiple LSTM layers.

The bidirectional LSTM network is another improvement of LSTM. Zhang et al. developed a bidirectional LSTM network for machine remaining life prediction [94]. Firstly, a one-layer perceptron network is designed for health indexing from raw input data. The deep neural network consists of two hidden LSTM layers, each of which is bidirectional. Both the forward path and backward path are computed independently, and their outputs are concatenated. The forward direction aims to discover the system variation pattern, while the backward direction is designed to smooth the predictions.

The other deep learning networks can also be adopted to improve the performance of LSTM. Convolutional operations were conducted on both the input-to-state and state-to-state transitions of the LSTM [106]. Different from the hybrid of CNN and RNN in the following section, this method not only preserved the advantages of LSTM but also incorporated time–frequency features [87]. Furthermore, there are also other enhancement technologies for LSTM. Chen et al. [88] integrated both those handcrafted features and automatically learned features for RUL prediction, and an attention mechanism was introduced to highlight the importance of different features.

In production environment, there are some special issues to address. Faced with the over-fitting problem in LSTM training, dropout techniques can be utilized [100]. The LSTM model should be adaptively optimized using techniques such as the resilient mean square back-propagation (RMSprop) method [83]. Maintenance in real-world applications is usually based on the RUL probability distribution function (PDF). However, the LSTM model is unable to obtain uncertainties. This problem is not thoroughly researched in the literature. The Monte Carlo simulation approach can be used to generate prediction uncertainties, and feed those uncertainties into LSTM model for training [83]. There are also cases when only part-life-cycle observation data are available. An online learning model was proposed for improved LSTM-based RUL prediction [82]. It uses online observation data to update parameters in real time.

(4) GRU methods

Gated recurrent unit (GRU) is a gating mechanism in recurrent neural network model [107]. It is a variant of LSTM with fewer parameters, because it synthesizes the input gate and forget gate of LSTM into a single reset gate. When adopting GRU for RUL, it can make full use of the historical state information of limited samples, and can effectively slow down the forgetting speed of important trend information [95].

It can be integrated with other neural networks to form a deep neural network, enabling it to extract features at different scale layers. Ren et al. [96] proposed a multi-scale dense gate recurrent unit network (MDGRU) for bearing remaining useful life prediction. A restricted Boltzmann machine network makes up the first two layers of MDGRU, which reduces the dimension of original features. Then, the output is fed into the following multi-scale time layer. This layer implements the embedding function that prevents the loss of information of the feature. Several skip-GRU layers employ dropout strategy, ReLU activation function, and Adam optimization algorithm to enhance the traditional GRU network. Finally, three dense layers implement ensemble learning and predict RUL value.

When constructing the input–output structure of the GRU network, there are two options: the sequence-to-sequence method and the sequence-to-one method. Chen et al. [97] developed a two-step solution for RUL prediction. In the first step, they applied kernel principal component analysis (KPCA) to extract nonlinear features. Then, they designed a gated recurrent unit (GRU) to predict RUL. Their experiment results show that sequence-to-one method is more suitable in their scenario, and GRU has advantages in both training performance and prediction accuracy.

The bidirectional RNN model is a common idea to extract features from time series [98]. Zhao et al. [99] proposed a local-feature-based gated recurrent unit (LFGRU) network, which combines handcrafted feature design with automatic feature learning. The raw sensory input is first divided into segments with a fixed window length. Then, tridomain features, including time, frequency, and time–frequency, are extracted from each local window. Bidirectional GRU is fed with the generated local feature sequence to learn representation features. Considering that information in the middle range of the sequence might be lost in a bidirectional GRU, they introduced a weighted feature averaging approach to highlight the impact of the middle local features. Two stacked fully connected dense layers, taking middle local features and learned representation features as input separately, formed the supervised learning of RUL prediction. A temporal self-attention mechanism was introduced into a novel bidirectional gated recurrent unit to predict RUL, where each considered time instance was assigned a self-learned weight [101].

#### 4.2.4. Convolutional Neural Network Model

Traditional machine learning methods are usually suitable for lower-dimensional features, while deep learning methods are suitable for high-dimensional features under the big data context. The CNN model is good at dealing with high-dimensional features using fewer parameters to achieve the same functionality and precision (Table 6). Therefore, it is a good choice for high-dimensional data, which is an outstanding characteristic in the context of RUL prediction.

The first attempt to apply CNNs for RUL prediction was conducted by Babu et al. [108]. Convolution and pooling filters in their approach are applied along the temporal dimension. Their model is constructed with two pairs of convolution layers and pooling layers, and one normal fully connected multi-layered perceptron network. To ensure equal contribution from all features across all operating conditions, a custom normalization mechanism was designed. Ren et al. [111] proposed a CNN architecture consisting of eight layers: three convolution layers, three pooling layers, one flatten layer, and one output layer. Considering the lack of time domain features, they designed a new feature extraction method called spectrum-principal-energy-vector to obtain the feature vector. As the RUL output of the CNN may not be continuous, they also proposed a linear regression method to smooth the forward prediction result.

To gain better knowledge of the input data with additional multi-scale feature information is an advantage of CNN model. Zhu et al. [109] proposed a multi-scale convolutional neural network (MSCNN) method for bearing RUL prediction. Wavelet transform (WT) is adopted to transform original data into a time frequency representation (TFR). Then, bilinear interpolation is used to reduce the data dimension. After that, the data are fed into the MSCNN for model training. Li et al. [110] proposed a deep convolutional neural network method for aero-engine RUL prediction. To select raw features, sensor data are processed using min–max normalization method, and samples are prepared in a time window manner. Based on this attempt, they further proposed a multi-scale CNN method to predict RUL of bearings [29]. The training and testing samples are prepared from measured data using the short-time Fourier transform (STFT) technique. The dropout technique is adopted to avoid the over-fitting problem. Furthermore, the leaky rectified linear units (leaky ReLU) technique [113] is adopted to optimize gradient vanishing and gradient diffusion problems. The difference between these two MSCNNs lies in the structure of the deep neural network. In the first MSCNN, a mixed layer is designed to accept features from the third convolutional layer and the second pooling layer. The output of the special mixed layer is then fed into the regression layer to perform RUL prediction. In the second MSCNN, three consecutive convolutional layers are designed for feature extraction. The features generated by the three layers are concatenated to preserve the diverse information and obtain features of multiple scales. Then, the concatenated features are fed into another convolutional layer to reduce dimensionality, followed by a fully connected layer. Li et al. [114] improved the efficiency of the MSCNN by setting different convolution kernel sizes in parallel at the same layer. In order to effectively identify the distinctions of different sensor data, a multi-scale convolutional attention network (MSCAN) [115] is proposed, where self-attention modules were first constructed to effectively fuse the input multisensor data.

Another important concept of CNN is the channel. Jiang et al. [112] proposed an enhanced CNN (ECNN) method for predicting the RUL of turbofan engines. The time series input of the CNN network has two channels, with the first channel being the earlier data and the second channel being the later data. This design takes advantage of temporal relationship between different channels. To train the networks, the data are preprocessed using mean removal and variance scaling normalization methods. For health indexing, the degradation point of an engine is chosen as half of the largest time cycle. After that, it degrades linearly. The network parameters are trained using an adaptive moment estimation (Adam) optimizer.

### 4.3. Deep Learning Methods for RUL Prediction

#### 4.3.1. Transfer Learning Method

A major challenge in data-driven prognostics is that it is often difficult to obtain a large number of failure samples. This situation arises for several reasons: (1) running until failure is not permitted for critical assets; (2) many failures occur slowly and follow a degradation path, which might take months or even years. To address the issue of data scarcity, transfer learning can be adopted by taking advantage of datasets in related domains or simulated datasets [116]. It has already made great progress in image, audio, and text processing scenarios. The basic idea is to train models on different but related source datasets first, and then tune the trained models using the target dataset. However, the data distribution in the source dataset may be different from that of the target dataset. In such cases, effective knowledge transfer is the key challenge in improving the performance of learning, enabling one to avoid dependence on the volume of failure samples. Based on the availability of sample labels, transfer learning can be divided into three categories: inductive transfer learning, transductive transfer learning, and unsupervised transfer learning [117]. Inductive transfer learning is employed when labeled data are available in a target domain. The transductive transfer learning is suitable for scenarios where labeled data are only available in the source domain. Furthermore, unsupervised transfer learning is leverage to address problems where there are no labeled data in both the source and target domains. When implementing a transfer learning approach, transfer learning can be conducted at four levels, according to the objects to be transferred: instance transfer, feature representation transfer, parameter transfer, and rational knowledge transfer.

Zhang et al. [118] proposed a transfer learning approach with bidirectional LSTM deep neural networks for RUL prediction. It belongs to inductive transfer learning and parameter transfer. They conducted a series of experiments on the C-MAPSS datasets [119] of different operation conditions. The experimental results showed that transfer learning is effective in most cases except when transferring knowledge from a dataset of multiple operating conditions to a dataset of a single operating condition, which led to the open problem of negative transfer learning. Sun et al. [120] presented a deep transfer learning (DTL) method. The deep learning model, trained by historical failure data, consists of stacked sparse auto-encoders (SAE), and one nonlinear regression layer. Then, three transfer strategies of weight transfer, feature transfer learning and weight update are used to transfer the SAE architecture to a new object scenario for RUL prediction. Da Costa et al. [121] proposed a new data-driven approach for domain adaptation in prognostics using long short-term neural networks (LSTM), where domains were composed of data with different fault modes and operating conditions. Zhu et al. [122] and Cao et al. [123] proposed transductive transfer learning for bearing RUL prediction under different conditions, which was conducted at the feature representation transfer level. Cheng et al. [124] implemented the same idea for bearing RUL prediction under multiple failure behaviors. Through adding failure behavior judgment, more refined transfer was implemented [125].

#### 4.3.2. Hybrid Deep Learning Method

In order to take advantage of different deep learning models, different models are integrated to generate hybrid model with higher accuracy. The most widely used paradigm is a two-stage hybrid model (Table 7). At the first stage, features are preprocessed using a deep neural network as a sparse auto-encoder. At the second stage, deep neural networks are trained to calculate the output from features generated at the first stage [126,127].

For the RUL prediction problem, Gao et al. [128] adopted stacked denoising auto-encoders (SDAE) to extract deep features. Then the deep features are fed into a support vector machine (SVM) to predict the RUL of integrated modular avionics (IMAs). Song et al. [129] proposed an auto-encoder–BLSTM hybrid method. It takes advantage of feature extracting capability of the auto-encoder and the temporal modeling power of BLSTM. Ren et al. [135] combined a deep auto-encoder and deep neural networks for bearings RUL prediction. They proposed a novel feature vector based on time–frequency–wavelet combined features to represent the bearing degradation process. Then, a deep auto-encoder was presented to compress the combined features without increasing the scale of the following nine-layer prediction DNN. This prediction DNN is featured with a special RUL normalization, named experience–max normalization. Then, the forward linear smoothing method is used to smooth the prediction results. Deutsch et al. [136] presented a DBN-FNN approach for predicting RUL of gears and bearings. The deep belief network, which has the advantages of self-taught feature learning capability, is made up of stacking RBMs. An FNN architecture is used for supervised fine-turning of RUL prediction. The optimal hyper-parameters of DBN-FNN structure were determined using a grid search method.

Different from general two-stage hybrid model, deep learning models can be mixed in a more complicated way. The hybrid of CNN and RNN is a common approach, where CNN is designed to extract local features, and RNN is designed to encode the temporal information [130,131,132,133]. Hinchi et al. [134] implemented the same idea, except that the bidirectional LSTM is replaced with a unidirectional LSTM model. Daroogheh et al. [137] proposed a novel hybrid architecture by integrating multiple models and neural network techniques. A mode-based particle filters (PFs) method is used to predict the propagation of health indicators. Then, a neural network based model is adopted to enhance the accuracy of the overall particle-filtering-based strategy. They considered three neural network paradigms: (1) MLP, (2) RNNs, and (3) WNNs. Experimental results show that all three paradigms are able to obtain reliable and accurate predictions. Furthermore, the performance is not dependent on the type of neural network structures, which validates both the accuracy and extendibility of the proposed hybrid method. Li et al. [138] designed a hybrid Elman–LSTM model for battery RUL prediction. They first implemented an empirical mode decomposition (EMD) algorithm to reconstruct battery capacity series. Then, the LSTM and Elman neural networks were combined to capture the battery capacity degradation features with increasing cycle number in the long term and to represent the capacity recovery at certain cycles in the short term.

#### 4.3.3. Ensemble Learning Method

Ensemble learning can be considered as a special type of hybrid learning. The performance of a deep learning model may be influenced by various factors, such as environmental uncertainties, different operational conditions, and the number of available sensors. A single model trained on data under certain situations may not generalize well to other situations. Ensemble learning provides a solution for this problem. An ensemble of multiple models is able to leverage the advantages of each individual model, and improve generalization capability.

Two main aspects to be considered when applying an ensemble learning method are the following: (1) how to design ensemble models; (2) how to fuse ensemble model outputs to generate final RUL value (Table 8).

Particle filter is often used as prognostic technique for estimating the evolution of the degradation state. It relies on analytical models of both degradation state evolution and measurement. The probabilistic relation between them is then used to update the prediction of the evolution of the degradation upon the acquisition of a measure within a Bayesian framework. Baraldi et al. [143] implemented a PF approach. They built the measurement model using ANNs.

Hu et al. [142] proposed an ensemble method composed of five models: three similarity-based interpolation approaches, one extrapolation-based approach, and one recurrent neural network approach. The K-fold cross validation (CV) technique was adopted to calculate the accuracy of the prediction models. Then, three weighted-sum formulation mechanisms were designed. First, the accuracy-based weighting mechanism is based on the CV accuracy. Second, the diversity-based weighting mechanism assigns higher weights to models with higher prediction diversity. Third, the proposed optimization-based weighting mechanism takes the advantages of both the first two and has proven to perform better in three cases: the 2008 IEEE PHM challenge problem, power transformer problem, and electric cooling fan problem.

Peel et al. [139] proved that the combination and filtering of models can yield a remarkable prediction performance. Their method won the IEEE GOLD category of the PHM’08 Data Challenge. The ensemble consisted of three models: a radial basis function (RBF) model with 15 hidden nodes and two MLP models, one with 75 hidden nodes and one with 100 hidden nodes. The ensemble models were selected using tournament heuristic algorithm. Then, the Kalman filter was adopted for fusing multiple neural network model predictions. The limitation of this approach is that it assumes the health of the system linearly degrades with usage, which may be divided into nonlinear health stage and degradation stage in real-world applications. A switching Kalman filter (SWF) approach [140,141] was proposed to overcome the limitation.

In order to select the most suitable models for an ensemble, it can be viewed as a multi-objective optimization problem. The two main objectives are accuracy and diversity. The accuracy objective quantifies the similarity between the output of a model and the real value, while the diversity objective measures the discrepancy between the outputs of multiple models. The accuracy objective improves the performance of each model, and the diversity objective improves the generalization performance.

Zhang et al. [147] proposed an ensemble learning method named multi-objective deep belief networks ensemble (MODBNE). It combines a multi-objective evolutionary algorithm with the traditional DBN technique to train a DBN ensemble. To obtain a population of candidate DBNs, they employed a widely used approach of MOEA/D [148]. The two conflicting objectives are maximizing accuracy and maximizing diversity. The solution space is defined in terms of DBN’s structural parameters and training parameters. After the candidate DBNs are trained, they are combined to generate the final prediction of RULs. The combination weights are optimized by differential evolution (DE) [149] algorithm. Rigamonti et al. [144] proposed an echo state networks (ESNs) ensemble approach for RUL prediction. The member ESN models are constructed using multi-objective differential evolution (DE) method. The two complementary objectives are cumulative relative accuracy (CRA) and Alpha-Lambda (α-λ) accuracy prognostic metrics. The CRA provides an average estimation of the RUL prediction relative error and, being a relative measure, tends to enlarge errors made at the end of the system’s life. On the other hand, the α-λ accuracy indicates how many times, on average, the RUL prediction falls within two relative confidence bounds. ESN outputs are fused using a dynamic local aggregation approach. Moreover, they also investigated the estimation uncertainty problem using MVE method, which is considered to be easily embedded within a local ensemble framework.

Different from most works that take operation conditions into consideration, Li et al. [145] proposed an ensemble-learning-based prognostic approach with degradation-dependent weights for RUL prediction. They employed an empirical model to determine the performance degrading stages. Then, the fusing weights for member algorithms are optimized in a stage-wise manner.

### 4.4. Ad Hoc Deep-Learning-Based RUL Prediction

#### 4.4.1. Multiple Operational Conditions Application

In practice, similar or identical engineered systems are exposed to different operational conditions for performing different tasks. Different operational conditions may significantly accelerate or decelerate the degradation process, which will affect the RUL of engineered systems [25,150,151]. Hence, it is challenging to finish the final step of applying deep-learning-based RUL prediction methods to a production environment.

The most straightforward method is to design a mechanism that is able to normalize different operation conditions. Bektas et al. [26] proposed a multi-regime normalization method to provide a common scale across different operation conditions. Based on normalized features, they presented a similarity-based deep learning method for RUL prediction.

Another idea is to take the operation conditions as input to a deep learning model. Huang et al. [25] developed a novel prognostic method based on bidirectional LSTM (BLSTM) networks to address the multiple operation conditions learning problem. Considering the complexity of health conditions, which are monitored as sensory data, their temporal hidden features are first extracted by a BLSTM. Then, operational conditions data and extracted features are taken as the input to another BLSTM. The basic component of a BLSTM is the common LSTM. The dropout technique and early stopping method were employed to relieve the over-fitting problem.

#### 4.4.2. Insufficient Labeled Data Application

Insufficient labeled data problem denotes that there is little historical data recorded with labels indicating the exact remaining useful life. There are two main reasons for this phenomenon. First, labeling work is labor-intensive and time-consuming. Second, most assets work in normal situations during their lifetime in production environment. In the case of mission-critical assets such as nuclear power plant, a single failure means a disaster.

The general method is semi-supervised learning (SSL), which is able to make use of both labeled and unlabeled data. The unlabeled data are fed into an unsupervised learning model, and the labeled data are fed into a supervised model. Yoon et al. [152] proposed an unsupervised variational auto-encoder (VAE) to translate the original space into a lower dimensional space. Then, an RNN model takes the output of the VAE as input and generates RUL. It is trained using labeled data. Listou Ellefsen et al. [153] proposed a five-layer deep network. The first layer is a restricted Boltzmann machine performing unsupervised learning on raw unlabeled input data. It uses a rectified linear unit (ReLU) as the activation function. For labeled data, the learned abstract features are fed into the subsequent deep architecture of two LSTM layers: one FNN layer and one output layer. The supervised learning deep network is trained through the truncated back-propagation through time (TBPTT) technique. Another method is to apply sufficient simulation data as the training data [116].

#### 4.4.3. Uncertainty of RUL Prediction

In real applications, estimated RUL usually varies widely due to the model parameters and noisy sensor data. Deterministic prediction values may not sufficient for RUL based operation decisions like maintenance. If the RUL prediction interval can be estimated, it would provide more information for operation decision.

Lacking uncertainty representation is currently a common disadvantage of deep learning methods. Zhao et al. [154] used a DBN-RVM fusing method to predict RUL of lithium-ion batteries. Based on the restricted Boltzmann machine (RBM), DBN is powerful in feature extraction and data reduction but lacks the capability of uncertainty representation. RVM has the capability for uncertainty representation but has low accuracy and poor stability in long-term prediction. By making full use of the advantages of these two techniques, their method demonstrates the effectiveness of prediction accuracy and reliability simultaneously. The bootstrap method was adopted to calculate the interval of RUL values calculated by the LSTM–FNN architecture [155]. Variational inference was employed to quantify the uncertainty of recurrent convolutional neural networks in prognostics, and a probabilistic RUL prediction result is obtained using Monte Carlo dropout [156]. Yang et al. [157] developed an improved dropout method based on nonparametric kernel density to improve the estimation accuracy of RUL. Furthermore, the Gaussian process regression (GPR) model is utilized to fit uncertainty quantification caused by battery capacity regeneration when using LSTM to predict RUL [158]. Assuming that the predicted RUL follows a normal distribution, a normal distribution output layer was designed to quantify uncertainty [159].

## 5. Challenge and Future Directions

**Figure 3:**
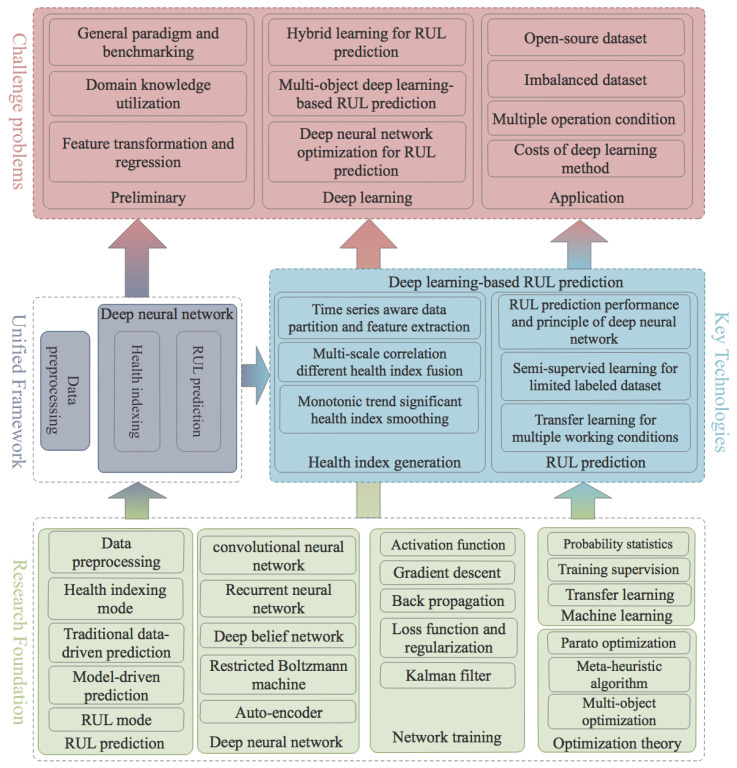
Future directions.

RUL prediction has always been important in industry and attracts a lot of attention in academia. Due to the complexity of the problem and limited by the current state of the art, RUL prediction has still not been widely used in real systems. Recently, deep learning technology has made breakthrough progress and has been widely applied in multiple areas. Naturally, it has also attracted attention of researchers in the RUL prediction field. This can be proved by the phenomenon of a dramatic increase in related works that use deep learning methods to predict RUL recently. However, it is still at its preliminary stage compared to other areas, taking speech recognition and LLM as examples. Figure 3 shows the overall research architecture of RUL prediction based on deep learning.

The research challenges and future directions of deep-learning-based RUL prediction are summarized as follows.

### 5.1. Unified Framework and Architecture

Most reviewed works are designed for specific assets or problems. Little is known about why and how these architectures and approaches have been designed and implemented. Hence, it is a challenge to design a general unified framework and provide a common paradigm to evaluate the validity of different methods. The future research directions are as follows.

#### 5.1.1. General Paradigm and Benchmarking

It is necessary to provide a general deep learning paradigm for RUL prediction. The performance of a deep learning model for RUL prediction should be evaluated in multiple dimensions. General benchmarking is needed to discover the advantages and disadvantages of different methods exactly, and help the development of deep-learning-based RUL prediction technology.

#### 5.1.2. Open-Source Dataset

Considering the huge model complexity and depth of deep learning model, the performance of RUL prediction is limited by the scale of training datasets. To publish large-scale open-source dataset is meaningful to promote the development of deep-learning-based RUL prediction technology.

### 5.2. Health Indicator Generation

A good health indicator (HI) often determines the accuracy and reliability of RUL prediction. Hence, it is the first challenge when considering the utilization of deep learning techniques for RUL prediction. The future research directions are as follows.

#### 5.2.1. Deep-Learning-Based Health Indexing

In order to generate efficient health indicators, deep learning methods are able to capture the key information in the process of degradation from raw signals, which does not rely heavily on the prior knowledge [160]. The dataset used to construct health indicators may be imbalanced and irregular under variable working conditions, so unsupervised deep learning techniques should be given more attention [161]. Furthermore, the variation of deep learning models should be considered to mine the hidden stationary and nonstationary information [162].

#### 5.2.2. Domain Knowledge Utilization

It is an advantage of deep learning that its performance does not strongly depend on domain knowledge. However, deep neural networks are still regarded as black boxes models currently. Their inner mechanisms are unexplainable. Domain knowledge can contribute to the success of applying deep learning on RUL prediction. It can be utilized to generate discriminative features which enables reducing the scale of the subsequent deep learning model. It can also be integrated into deep neural network training, such as regularization term designing for higher generalization capability.

#### 5.2.3. Feature Transformation and Regression

The goal of feature transformation and regression is to generate a health indicator satisfying the requirements of monotonicity, trendability, and prognosability. It is a complicated task to ensure the obtaining of the maximum of meaningful information from raw signals. However, efficient feature transformation and regression can not only improve the accuracy of RUL prediction, but also reduce the costs of complex deep learning models.

### 5.3. Deep-Learning-Based RUL Prediction

RUL prediction is the essential process that calculates the RUL value from the health indicator or even raw input data. The first challenge is to evaluate the performance of RUL prediction with different deep neural networks and analyze the principle behind them. Then, different supervised learning methods and transfer learning technologies can be adopted as a complement to solve specific problems. When the models are chosen, the second challenge is to optimize them for efficient RUL prediction. The future research directions are as follows.

#### 5.3.1. Hybrid Learning for RUL Prediction

There is a consensus that the performance of RUL prediction can be improved by hybrid learning methods. The most widely used hybrid learning method is ensemble learning. The models composing the ensemble will generate RUL values in parallel. Then, these RUL values are assigned with weights under special mechanism and produce the final RUL value. Another category of hybrid learning method is to take advantage of different deep neural networks in general manner. A classic combination is to utilize restricted Boltzmann machine or auto-encoder at the health indexing stage, and recurrent neural network or convolutional neural network at the RUL prediction stage. Moreover, hybrid learning enables extracting more features from different deep neural network models and layers of neural network, which in turn creates potential for getting more accurate RUL value.

#### 5.3.2. Deep Neural Network Optimization for RUL Prediction

Accompanied by deep learning, the training of deep neural networks is also a challenging problem. Problems of traditional deep learning also exist in RUL prediction scenarios, including over-fitting, under-fitting, gradient explosion, and gradient disappearance. Hyperparameter tuning is another direction worthy of deep research. Currently, hyperparameters are mostly manually designed, which is overly dependent on manual experience and may not yield sufficient accuracy. This can be optimized by meta-heuristic algorithms such as the genetic algorithm and particle swarm optimization.

### 5.4. Real-World Applications

The final goal of prediction is indeed to estimate the RUL of real-world assets. When dealing with real-world environments, the challenges primarily lie in three areas: source data collection, operating condition identification, and model deployment. Some potential future research directions related to these challenges are presented here.

#### 5.4.1. Imbalanced Dataset

The imbalanced dataset is a widely existing problem in production environment. The amount of fault data is much less than that of health data. Enhanced deep learning methods of unsupervised learning, semi-supervised learning, and transfer learning can be proposed to address this issue.

#### 5.4.2. Multiple Operation Condition

Currently, most works assume that assets work in constant operation condition, which is not the truth in real production environments. The gap between ideal experimental working conditions and dynamic operation conditions should be covered.

#### 5.4.3. Cost of Deep Learning Method

Within this survey, few works concern the costs, especially for the computations, of various deep learning models. This may stem from the difficulty of comparing models across different hardware and asset types. The costs may also be disregarded under the assumption of an ideal experimental environment. However, accurate RUL prediction should be carried out in a time-critical manner in order to finally realize the benefit from deep-learning-based RUL prediction.

#### 5.4.4. Multi-Objective Deep-Learning-Based RUL Prediction

Generally, the goal of deep-learning-based RUL prediction is accuracy. However, it is hard to obtain the most accurate model, and the accuracy may change with application scenario even for the same model. It can be formalized as multi-objective optimization problem. Taking accuracy and diversity difference as two conflicting objectives, it would be able to obtain Pareto-optimal results.

## 6. Conclusions

Deep-learning-based RUL prediction is comprehensively surveyed. All related works are reviewed in three dimensions. Firstly, a unified framework is proposed to analyze relative works and guide future method design. It divides the overall process into three stages of data preprocessing, health indicator generation, and RUL prediction. Then, the details of prediction are compared from the perspective of different deep learning models, including the auto-encoder, the restricted Boltzmann machine, the deep belief network, the recurrent neural network, and the convolutional neural network. Thirdly, the literature is reviewed from the perspective of specific problems, including transfer learning, the multiple operational conditions method, the insufficient labeled data method, hybrid deep learning, ensemble learning, and the uncertainty of RUL prediction. Based on the systematic review, the key technologies and some challenging problems are summarized.

## Figures and Tables

**Table 1:** Acronyms used in the survey.

Notation	Description
TBM	Time-based maintenance
CBM	Condition-based maintenance
HI	Health indicator
DNN	Deep neural network
ANN	Artificial neural network
AE	Auto-encoder
SDA	Stacked denoising Auto-encoder
SAE	Stacked sparse Auto-encoder
EAE	Enhanced Auto-encoder
RBM	Restricted Boltzmann machines
DBN	Deep belief network
CDBN	Continuous deep belief network
FNN	Feed-forward neural network
RNN	Recurrent neural network
RNN-ED	RNN encoder–decoder
ESN	Echo state network
LSTM	Long short-term memory
GRU	Gated recurrent unit
CNN	Convolutional neural network
MSCNN	Multi-scale convolutional neural network
PCA	Principle component analysis
KPCA	Kernel principal component analysis
MA	Moving average method
EWMA	Exponentially weighted moving average
GWO	Grey wolf optimizer
SOM	Self-organizing map
CWT	Continuous wavelet transform
GPR	Gaussian process regression
ELM	Extreme learning machines
HELM	Hierarchical extreme learning machines
ACO	Ant colony optimization
GA	Genetic algorithm
BPTT	Back-propagation through time
SSL	Semi-supervised learning
ReLU	Rectified linear unit
GBR	Gradient-boosting regression

**Table 2:** Comparison between related survey works.

	PHM	RUL	TDD	SNN	DNN
Schwabacher et al. [14]		✓		✓	
Si et al. [8]		✓	✓		
Liu et al. [15]	✓		✓		
Nash et al. [16]	✓				✓
Zhao et al. [17]	✓				✓
Khan et al. [18]	✓				✓
Zhao et al. [19]	✓				✓
Remadna et al. [20]		✓			✓
Wang et al. [21]		✓			✓

**Table 3:** Health indexing technologies.

	Transformation and Selection	Fusion and Regression
Baraldi et al. [39]	Binary Differential Evolution	Auto-Associative Kernel Regression
Guo et al. [40]	CNN	3σ rule
Li et al. [41]	KPCA	Exponentially weighted moving average
Zhao et al. [42]	Enhanced autoencoder, SOM network	Grey wolf optimizer
Yoo et al. [42]	CWT, CNN	Gaussian process regression
Xia et al. [43,44]	SDA	shallow ANN
Senanayaka et al. [45]	CWT, sparse autoencoder	LSTM
Guo et al. [46]	Related-similarity feature approach	RNN
Gugulothu et al. [47,48]	RNN-ED	Masking vector, Delta vector
Hasani et al. [49]	Auto-encoder	Moving average filter
Chen et al. [50]	CNN, bidirectional GRU	GRU

**Table 4:** RBM methods.

	Field	Structure
Ma et al. [61]	Bearings, turbine engine	Stacked RBMs followed by an discriminative fine-tuning layer
Wang et al. [62]	Material removal rate	Stacked RBMs followed by an feed-forward three layers perceptron network
Shao et al. [63]	Rolling bearing	CDBN
Liao et al. [59]	Bearings	Enhanced RBM with SOM method for feature fusion

**Table 5:** RNN methods.

	Field	Structure
Tse et al. [69]	Industrial machines	The output node is feedback loop linked to extra input nodes.
Malhi et al. [70]	Rolling bearing	The output node is feedback loop linked to extra input nodes.
Tian et al. [71]	Gearbox	Extended RNN
Liu et al. [72]	Lithium-ion battery	Adaptive RNN
Heimes et al. [27]	PHM 08 Challenge dataset	RNN
Peng et al. [73]	Turbofan engine	Modified ESN
Morando et al. [74,75]	Proton Exchange Membrane Fuel Cell	ESN
Zheng et al. [76]	C-MAPSS dataset, PHM 08 Challenge dataset, Milling dataset	LSTM
Liu et al. [77]	PEMFC	LSTM
Zhang et al. [78], Chemali et al. [79], Li et al. [80], Park et al. [81], Zhou et al. [82], Zhang et al. [83]	Lithium-ion battery	LSTM
Dong et al. [84]	Jet engines	LSTM
Yuan et al. [85]	Aero engines	LSTM
Wang et al. [86]	Rolling bearing	LSTM
Xiang et al. [87]	Aero engines	MCLSTM
Chen et al. [88]	C-MAPSS dataset	LSTM
Hsu et al. [89]	C-MAPSS dataset	Stacking two LSTM layers
Lima et al. [90]	Hard disk drivers	Stacking two LSTM layers and one fully connected layer
Zhang et al. [91]	Gas turbine engine	Deep LSTM
Zheng et al. [92]	Equipment system	Deep LSTM
Zhao et al. [93]	Tool wear	Stacking multiple LSTM layers
Zhang et al. [94]	C-MAPSS dataset	Bidirectional LSTM
Zhou et al. [95]	Bearing	CMGRU
Ren et al. [96]	Bearing	MDGRU
Chen et al. [97]	C-MAPSS dataset	GRU
She et al. [98]	Bearing	BiGRU
Zhao et al. [99]	Tool wear	LFGRU
Wu et al. [100]	C-MAPSS dataset	deep LSTM
Zhang et al. [101]	Turbofan engine	BiGRU

**Table 6:** CNN methods.

	Field	Structure
Babu et al. [108]	C-MAPSS dataset, PHM 08 Challenge dataset	CNN
Zhu et al. [109]	Bearing	MSCNN
Li et al. [110]	C-MAPSS dataset	DCNN
Li et al. [29]	PHM 2012 Challenge dataset	MSCNN
Ren et al. [111]	Bearing	CNN
Jiang et al. [112]	C-MAPSS dataset	ECNN

**Table 7:** Hybrid methods.

	Field	Structure
Gao et al. [128]	IMA	SDAE, SVM
Song et al. [129]	Turbofan Engine	Autoencoder–BLSTM
Zhao et al. [130]	CNC machine	CNN, BLSTM
An et al. [131]	Milling tool	CNN, LSTM
Liu et al. [132]	Turbofan Engine	CNN, BGRU
Ren et al. [133]	Lithium-ion batteries	CNN, LSTM
Hinchi et al. [134]	Rolling element bearing	CNN, LSTM
Ren et al. [135]	Bearings	Deep autoencoder, DNN
Deutsch et al. [136]	Gears, bearings	DBN, FNN
Daroogheh et al. [137]	Gas turbine engine	PFs, MLP, RNNs, WNN
Li et al. [138]	Lithium-ion batteries	LSTM, Elman neural networks

**Table 8:** Ensemble learning methods.

	Field	Models	Feature Fusion
Peel et al. [139]	PHM 2008 Dataset	RBF, MLP	Kalman filter
Lim et al. [140,141]	PHM 2008 Dataset	MLPs	Switching Kalman filter
Hu et al. [142]	PHM 2008 Dataset, power transformer, electric cooling fan	RVM, SVM, exponential fitting, quadratic fitting, RNN	Accuracy-based weighting, diversity-based weighting, optimization-based weighting
Baraldi et al. [143]	Crack propagation	ANNs	Particle filter
Zhang et al. [75]	Rolling element bearing	ANNs	Dynamically weights updating
Rigamonti et al. [144]	C-MAPSS dataset, Cutting knives	ESNs	Dynamically local aggregation
Li et al. [145]	Aeroengine Bearings, Aircraft engines	RS, ES, SS, QB, RNN	Degradation-dependent weighting
Xia et al. [146]	C-MAPSS dataset	CNN-BLSTM	Weighted average method

## Data Availability

Not applicable.
